# The Processing Cost of Scrambling and Topicalization in Japanese

**DOI:** 10.3389/fpsyg.2016.00531

**Published:** 2016-04-19

**Authors:** Satoshi Imamura, Yohei Sato, Masatoshi Koizumi

**Affiliations:** ^1^Faculty of Oriental Studies, Research Centre for Japanese Language and Linguistics, University of OxfordOxford, UK; ^2^Department of Linguistics, Faculty of Arts and Letters, Tohoku UniversitySendai, Japan

**Keywords:** syntactic complexity, information structure, production frequency, processing cost, scrambling, topicalization, Japanese, sentence comprehension

## Abstract

This article presents two reading comprehension experiments, using the sentence correctness decision task, that explore the causes of processing cost of Japanese sentences with S_NOM_O_ACC_V, S_TOP_O_ACC_V, O_ACC_S_NOM_V, and O_TOP_S_NOM_V word orders. The first experiment was conducted in order to see if either syntax or frequency plays a significant role in the processing of these sentences. The results of the first experiment have shown that both the structure-building process and frequency directly affect processing load. We observed that there was no difference in processing cost between S_NOM_O_ACC_V and S_TOP_O_ACC_V, both of which are easier to process than O_ACC_S_NOM_V, which is in turn easier to process than O_TOP_S_NOM_V: S_NOM_O_ACC_V = S_TOP_O_ACC_V < O_ACC_S_NOM_V < O_TOP_S_NOM_V. This result is the mixture of the two positions. Specifically, the structure building cost of S_TOP_O_ACC_V was neutralized by its high frequency. The aim of the second experiment was to investigate the interaction between syntactic structure, frequency, and information structure. The results showed that the processing cost of O_ACC_S_NOM_V was facilitated by given-new ordering, but S_NOM_O_ACC_V, S_TOP_O_ACC_V, and O_TOP_S_NOM_V were not. Thus, we can conclude that information structure also influences processing cost. In addition, the distribution of informational effects can be accounted for by Kuno's (1987, p. 212) Markedness Principle for Discourse Rule Violations: S_NOM_O_ACC_V and S_TOP_O_ACC_V are unmarked/canonical options, and as such are not penalized even when they violate given-new ordering, O_ACC_S_NOM_V is penalized when it does not maintain given-new ordering because it is a marked/non-canonical option, and O_TOP_S_NOM_V is penalized even when it obeys given-new ordering possibly because more specific contexts are needed. Another reason for the increased processing cost of O_TOP_S_NOM_V is a garden path effect; upon encountering O_TOP_ of O_TOP_S_NOM_V, the parser preferentially (mis)interpreted it as S_TOP_ due to a subject-before-object preference. The revision of the interpretation may be the cause of the high processing cost observed in O_TOP_S_NOM_V.

## Introduction

A language has various mechanisms for expressing the same propositional meaning. In Japanese, not only the canonical transitive sentence (1a) but also a scrambled sentence (1b) can describe the event *Taro chased Jiro*. There is no difference in the propositional meaning between SOV and OSV in (1). Moreover, the nominative case marker *GA* or accusative case marker *O* can be replaced by the topic marker *WA* with no change in propositional content as shown in (1c) and (1d).

(1) a. Taro-ga       Jiro-o       oikaketa. S_NOM_O_ACC_V
         Taro-NOM Jiro-ACC chased
         “Taro chased Jiro.”
    b. Jiro-o       Taro-ga       oikaketa. O_ACC_S_NOM_V
         Jiro-ACC Taro-NOM chased
         “Taro chased Jiro.”
    c. Taro-wa    Jiro-o       oikaketa. S_TOP_O_ACC_V
         Taro-TOP Jiro-ACC chased
         “Taro chased Jiro.”
    d. Jiro-wa    Taro-ga       oikaketa. O_TOP_S_NOM_V
         Jiro-TOP Taro-NOM chased
         “Taro chased Jiro.”

Yet, numerous studies on Japanese have observed that non-canonical sentences like (1b) incur higher processing costs than canonical sentences like (1a) (Chujo, 1983; Miyamoto and Takahashi, 2002, 2004; Tamaoka et al., 2005; Koizumi and Tamaoka, 2010). There are three possible explanations for this difference. The first possibility is that complex structures require heavier processing costs than simple structures do because of a language–universal parsing algorism (e.g., Frazier and Fodor, 1978; Frazier, 1987; Miyamoto and Takahashi, 2004). For example, according to Gibson (1998), processing non-canonical structures requires greater memory resources than processing canonical structures because the former violates the locality principle. Under this explanation, the cause of difficulty for scrambling is related to working memory resources. The main point is that, the mechanism of the parser is not influenced by its experience. The second possibility derives from frequency factors (MacDonald et al., 1994; Trueswell et al., 1994; Trueswell, 1996; Reali and Christiansen, 2007; Arnon and Snider, 2010). According to this position, the parser anticipates the following constituents based on its experience. Therefore, the more frequently a construction is used, the easier it becomes to process. Let us consider Japanese scrambling based on this position. As Imamura and Koizumi (2011), Kuno (1973), and Miyajima (1964) observed, the frequency of OSV is extremely low compared to that of SOV. Taken together, the parser may have greater difficulty in processing OSV than in processing SOV because of its low frequency. The third possibility is pertinent to information structure. Some scholars have claimed that the processing costs observed in previous studies were caused by Discourse Rule Violations (Kaiser and Trueswell, 2004; Roland et al., 2012). In experiments, sentences are often shown to participants without discourse context. Generally speaking, however, there are few cases where isolated sentences are used in natural discourse. Therefore, such an unnatural environment in experiments may be the cause of difficulty in processing non-canonical sentences. Specifically, in Finnish, Kaiser and Trueswell (2004) observed that the processing cost of scrambling was alleviated by discourse context. Thus, the processing cost of scrambling in Japanese may also be influenced by discourse context. Which hypothesis, then, provides the best explanation of the processing cost of scrambling in Japanese? Moreover, no previous studies have investigated the processing cost of the topicalization as in (1d). Thus, topic marker *WA* is taken into consideration. Which hypothesis can account for the processing cost of the sentences with a topic marker *WA* like (1c) and (1d)? In this paper, we will explore these questions based on two experiments of sentence comprehension.

This paper is organized as follows. In Section Previous Studies, we provide an overview of previous studies regarding scrambling, topicalization, and processing cost. In Section Experiment 1, we describe the details of the first experiment, which was conducted to examine the influence of syntactic structure and frequency on the processing of scrambling and topicalization. In Section Experiment 2, we present the second experiment, which took information structure into account. Additionally, we attempt to explain the results based on Kuno's (1987, p. 212) Markedness Principle for Discourse Rule Violation. In Section General Discussion, we discuss the results of the first and second experiments, and in Section Conclusion, we draw the conclusion from our findings.

## Previous studies

### Processing cost

#### Complexity

Numerous studies have demonstrated that a complex structure requires higher processing costs than a simple structure (Frazier and Fodor, 1978; Frazier, 1987; Pritchett and Whitman, 1995; Gibson, 1998, 2000; Miyamoto and Takahashi, 2004; Grodner and Gibson, 2005). Specifically, Gibson (1998, 2000) and Grodner and Gibson (2005) asserted that a capacity limitation for working memory is the main source of difficulty pertinent to complex structures. For example, it is a well-established finding that an object relative clause (OR) like (2b) incurs a higher processing cost than a subject relative clause (SR) like (2a) (e.g., Ford, 1983; King and Just, 1991; Staub, 2010)^1^. Grodner and Gibson (2005) maintained that the cause of this difference derives from the memory cost of *who*. In (2a), the parser does not need to keep *who* for a long time because *who* is locally linked with *sent*, which is adjacent to *who*. Thus, the burden on working memory is low. In contrast, in (2b), in order to integrate *who* with *sent*, the parser must cross *the photographer* and access the antecedent of *who*. During that process, the parser must store *the photographer* in its working memory for a long time. Hence, the processing cost is high. Thus, complexity is one of the sources of processing cost.

(2) a. SR: The reporter who sent the photographer to the editor hoped for a story.
    b. OR: The reporter who the photographer sent to the editor hoped for a story.
         (Grodner and Gibson, 2005, p. 266)

#### Frequency

It has been demonstrated that frequency has a strong influence on processing cost (MacDonald et al., 1994; Trueswell et al., 1994; Trueswell, 1996; Reali and Christiansen, 2007; Gennari and MacDonald, 2009; Arnon and Snider, 2010). According to experience-based theory, human parsers learn how to process sentences efficiently based on their experiences. Hence, frequent words, phrases, and constructions are processed more easily than infrequent expressions. For example, although (3a) is an OR and (3b) is an SR, (3a) incurs lower processing costs than (3b) does. This fact can be explained by supposing that an OR followed by a 1^st^ or 2^nd^ person pronoun subject is a frequently used pattern. Since the frequency of (3a) is high, the processing of the OR is facilitated. Most significantly, there is no syntactic difference between (2) and (3), but the processing costs of the SR and OR are reversed: the OR is more difficult to process than the SR in (2), but the OR is easier to process than the SR in (3). Thus, complexity cannot explain this difference, whereas frequency can.

(3) a. The consultant that you called emphasized the need for additional funding.
    b. The consultant that called you emphasized the need for additional funding.
         (Reali and Christiansen, 2007, p. 9)

#### Discourse context

Several studies have shown that discourse context has an effect on processing cost (Hoeks et al., 2002; Kaiser and Trueswell, 2004; Mak et al., 2008; Brown et al., 2012; Roland et al., 2012). Discourse-based accounts assume that the sentences employed in previous experiments are unnatural because they are isolated from discourse contexts. Thus, within an appropriate context, the processing cost related to non-canonical word orders may disappear. Kaiser and Trueswell (2004) performed a self-paced reading task in Finnish in order to see if discourse context could facilitate a lower processing cost of non-canonical word order. For instance, (4a) introduces *hiiren* “mouse” or *jäniksen* “hare” as a discourse context for (4b). When *hiiren* is mentioned in (4a), (4b) becomes OnewVSgiven because the subject is given information. On the other hand, when *jäniksen* is referred to in (4a), (4b) becomes OgivenVSnew because the object is given information. Kaiser and Trueswell observed that the reading times for OgivenVSnew were faster than those of OnewVSgiven. Their experiment reinforces the idea that the processing cost pertinent to non-canonical word order can be alleviated by discourse contexts.

(4) a. *Lotta  etsi           eilen        sieniä          metsässä*.
         Lotta   looked.for yesterday mushrooms forest.in
         “Lotta looked for mushrooms in the forest yesterday.”
         *Hän           huomasi heinikossa hiiren/jäniksen*
         s/he.NOM noticed    grass-in      mouse.ACC/hare.ACC
         joka liikkui          varovasti eteenpäin
         that  was.moving carefully    forward
         “In the grass, s/he noticed a mouse/hare
         that was moving forward carefully.”
    b. *Jänistä         seurasi   hiiri                ja  linnut*
         *lauloivat*
         Hare.PART   followed mouse.NOM and birds
         were.singing
         “A hare followed the mouse and birds were singing.”
    (Adapted from Kaiser and Trueswell, 2004, p. 12)

### Scrambling

Syntactically, it has been generally assumed that O_ACC_S_NOM_V includes a movement of the direct object from the VP-internal domain to the beginning of a sentence (Saito and Hoji, 1983; Saito, 1985, 2009; Miyagawa, 2001, 2003, 2010). This type of movement is termed “scrambling.” It is important to note that scrambling does not influence the propositional meaning. Thus, both the basic word order sentence (5a) and the scrambled word order sentence (5b) convey the same propositional meaning: *Taro ate pizza*.

(5) a. SOV: Taroo-ga     piza-o         tabeta.
                  Taro-NOM pizza-ACC ate
                  “Taro ate pizza.”
    b. OSV: piza-o         Taro-ga       tabeta.
                  pizza-ACC Taro-NOM ate
                  “Taro ate pizza.”

In SOV, it is a standard analysis that the *GA*-marked subject is base-generated in the VP-internal position and is moved to TP-Spec as shown in (6) (Miyagawa, 1989; Kishimoto, 2001). However, there is disagreement over the subject position in OSV sentences because surface positions do not provide enough information to determine the syntactic position of the subject. Saito (2003) supposes that both the subject and the object are generated in the VP-internal position and move to the VP-external position as shown in (7a). In contrast, Miyagawa (2001) argues that the subject stays in its VP-internal position whereas the object moves to TP-Spec as shown in (7b).

(6) S_NOM_O_ACC_V [_TP_ S_i_ [_VP_ t_i_ O V]]

(7) O_ACC_S_NOM_V
a.  [_TP_ O_j_ S_i_ [_VP_ t_i_ t_j_ V]]
b.  [_TP_ O_i_ [_VP_ t_i_' S t_i_V]]

Koizumi and Tamaoka (2010) conducted a sentence comprehension experiment in order to situate the subject position in OSV orders. Their design is based on the position of VP-adverbs, which are base-generated within VP. The reaction times increased when a VP-adverb is placed outside of its VP-internal position due to scrambling. Thus, if (7a) is correct, the reaction times for A(dverb)OSV and OASV will be longer than those of OSAV because VP-adverbs in AOSV and OASV are considered to be outside of their VP-internal positions. On the other hand, if (7b) is right, the reaction times for AOSV will be longer than for OASV and OSAV because only in AOSV is the VP-adverb located outside of VP^2^. The results of the experiment supported (7b). Therefore, in this paper we assume that the structure of O_ACC_S_NOM_V is the same as that in (7b).

Several studies have contended that scrambling is a discourse-related option (e.g., Kuno, 1978; Ishii, 2001; Kaiser and Trueswell, 2004; Imamura, 2014, 2015). In particular, Kuno (1978, p. 54) proposed that word order choice in Japanese is determined by given-new ordering, which means that given information is provided at the beginning and new information toward the end of a sentence. According to his proposal, OSV should be selected when the direct object is older information than its subject. Based on corpus analysis, Imamura (2014, 2015) demonstrated that scrambled direct objects tend to be given information in OSV orders. Moreover, according to Ishii (2001), the acceptability of the scrambled transitive sentence is increased when its object is attached with the demonstrative *sono*, or “that.” In fact, (8b) is more natural than (8a), which sounds slightly awkward without a prior context to make it clear that the bare noun object refers to a discourse-given entity. To summarize the above discussion, the O in OSV sequences seems to be related to given information.

(8) a. # Okane-o   dare-ga       nusun-da-no?
         money-ACC who-NOM steal-PAST-Q
    b. Sono okane-o        dare-ga       nusun-da-no?
         that    money-ACC who-NOM steal-PAST-Q
         “Who stole that money?” (Adapted from Ishii, 2001, p. 97)

In sentence processing, numerous studies on many languages have demonstrated that the processing cost of scrambled word order is higher than that of canonical word order. Frazier and Flores d'Arcais (1989) provide examples from Dutch, Erdocia et al. (2009) from Basque, Rösler et al. (1998) and Weyerts et al. (2002) from German, Sekerina (2003) from Russian, Tamaoka et al. (2011) from Sinhalese, and Kim (2012) from Korean. In Japanese, it has been reported in sentence comprehension that the reaction times for scrambled word orders were longer than those for canonical word order (Chujo, 1983; Miyamoto and Takahashi, 2002; Tamaoka et al., 2005; Koizumi and Tamaoka, 2010). All of these studies support the opinion that scrambled sentences are more difficult to process than canonical sentences. What then, is the cause of the processing cost for scrambling? We will clarify this issue from the viewpoints of complexity, frequency, and discourse.

### Topicalization

In Japanese syntax, it has been proposed that the topic phrase *XP-WA* has a more specialized position than TP-Spec (Kuno, 1973; Shibatani, 1990; Tateishi, 1994). Among others, Shibatani (1990) presumes that *WA*-marked phrases are external to the S(=TP) and dominated by S'. For instance, although there is a structural difference between (9a) and (9b), both *zoo* “elephant” in (9a) and *tori* “bird” are considered to be placed in the S' position. Put simply, *XP-WA* has a specialized position at S'.

(9)  a.  [_S‘_   Zoo-wa            [_S_       hana-ga       nagai]
         elephant-TOP             nose-NOM long
         “An elephant's nose is long.”
    b.  [_S‘_ Tori-wa              [_S_ [e]  tamago-o    umu]]
         bird-TOP                    egg-ACC    lay
         “A bird lays eggs.”
         (Shibatani, 1990, p. 274)

Following the hypothesis that the topic phrase *XP-WA* is structurally higher than TP, we presuppose that *XP-WA* belongs to the CP domain. Hence, the basic structure of S_TOP_O_ACC_V ([_CP_ S_TOPi_ [_TP_ t_i_' [_VP_ t_i_ O V]]]) is considered to be more complex than that of S_NOM_O_ACC_V.

Next, we examine how the structure with a topicalized object (O_TOP_S_NOM_V) is composed. Since we suppose that *XP-WA* belongs to the CP domain, O_TOP_ is also placed in the CP domain. Moreover, Kuroda (1987) claimed that *WA*-marked objects in O_TOP_S_NOM_V are derived from movement. Taken together, we assume that the derivation of O_TOP_S_NOM_V involves a topicalization movement in addition to a scrambling movement ([_CP_ O_TOPi_ [_TP_ t_i_” [_VP_ t_i_' S t_i_V]]]), and hence is more complex than that of O_ACC_S_NOM_V, which only involves scarmbling.

In terms of frequency, several studies have shown that the frequency of OSV is much lower than that of SOV (Miyajima, 1964; Kuno, 1973; Imamura and Koizumi, 2011). Imamura and Koizumi (2011) also revealed that, among the four types of sentences in question, S_TOP_O_ACC_V (82.5%) occurs much more frequently than S_NOM_O_ACC_V (14.7%) but there is no large difference in frequency between O_TOP_S_NOM_V (1.8%) and O_ACC_S_NOM_V (1%) in Japanese novels. The higher frequency of S_TOP_O_ACC_V than S_NOM_O_ACC_V may be partly because it produces cohesion in discourse by maintaining the same topic across sentences^3^.

In processing, no previous studies have attempted to investigate the processing cost of topicalization in Japanese. Almost all previous experiments on OSV have focused on O_ACC_S_NOM_V rather than on O_TOP_S_NOM_V. Therefore, our study is the first to examine the processing cost pertinent to a topicalized phrase with the topic marker *WA*. In this paper, we shed further light on topic constructions through the results of experiments we conducted based on complexity, frequency, and discourse.

Approval for the study was obtained from the Ethics Committee of Graduate School of Arts and Letters, Tohoku University.

## Experiment 1

### Predictions

The aim of the first experiment was to investigate the effects of syntactic structure and frequency on the processing cost of S_NOM_O_ACC_V, S_TOP_O_ACC_V, O_ACC_S_NOM_V, and O_TOP_S_NOM_V word orders, using a sentence comprehension experiment. A syntactic account predicts that non-canonical structures are more difficult to process than canonical structures because the former are more demanding in terms of working–memory load. Thus, both O_ACC_S_NOM_V and O_TOP_S_NOM_V are expected to be processed with more difficulty than S_NOM_O_ACC_V and S_TOP_O_ACC_V. Moreover, S_TOP_O_ACC_V is considered to be more complex than S_NOM_O_ACC_V, and O_TOP_S_NOM_V seems to be more complex than O_ACC_S_NOM_V because *WA*-marked constituents are supposed to be placed in the CP domain. Taken together, a syntactic explanation anticipates that S_NOM_O_ACC_V is easier to process than S_TOP_O_ACC_V, which in turn is easier to process than O_ACC_S_NOM_V, which, finally, is easier to process than O_TOP_S_NOM_V: S_NOM_O_ACC_V < S_TOP_O_ACC_V < O_ACC_S_NOM_V < O_TOP_S_NOM_V.

In terms of frequency, S_TOP_O_ACC_V occurs more frequently than S_NOM_O_ACC_V does. In addition, the frequency of SOV is higher than that of OSV. Taken together, a frequency-based account expects that the processing cost of O_ACC_S_NOM_V and O_TOP_S_NOM_V is higher than that of S_NOM_O_ACC_V, whose processing cost in turn is higher than S_TOP_O_ACC_V: S_TOP_O_ACC_V < S_NOM_O_ACC_V < O_ACC_S_NOM_V = O_TOP_S_NOM_V.

### Method

#### Participants

Forty-eight Japanese graduate and undergraduate students (23 males and 25 females) at Tohoku University participated in the first experiment. Their average age was 20.4 years.

#### Materials

Our experiment used a 2 × 2 factorial design with the scrambling factor (+/−scrambling) and the topicalization factor (+/−topicalization) as variables. Thus, as in (10), the first experiment was based on the four following experimental conditions:

(10) Experimental Conditions
a.  S_NOM_ O_ACC_V   (−scrambling, −topicalization)
b.  S_TOP_ O_ACC_V    (−scrambling, +topicalization)
c.  O_ACC_ S_NOM_V   (+scrambling, −topicalization)
d.  O_TOP_ S_NOM_V   (+scrambling, +topicalization)

Ninety-six transitive sentences were employed for the sentence correctness decision task, which calculated the reading time of a whole target sentence. Half of them were semantically correct (or plausible) sentences and the others were incorrect (or implausible) sentences. After they were shuffled in a Latin Square Design and split into four lists, 24 filler sentences were added. Only the reaction times and error rates for correct sentences were within the scope of our statistical analysis. The lexical material of the sentences was controlled in length and frequency. All the subjects and objects employed were proper nouns, all of which were three morae and consisted of two Chinese characters. The frequency of the proper nouns was controlled within the pair of a subject and object. Moreover, none of the nouns or verbs were repeated in order to avoid priming effects. An example is shown in (11). Here, both the subject *Satoo* and the object *Suzuki* are three-morae nouns. In other words, the subject and object do not differ in length. Further, both *Satoo* and *Suzuki* are proper nouns, and there is no difference in animacy between them since they are both human beings. Note that *Satoo* and *Suzuki* are family names. Thus, they are gender–neutral.

(11) Satoo-ga     Suzuki-o       hometa.
         Sato-NOM Suzuki-ACC praised
         “Sato praised Suzuki.”

With regard to incorrect sentences, they are unacceptable due to semantic type mismatches (selectional restriction violations). In (12), for instance, the transitive verb *nomu* “drink” requires something to drink, but the direct object is a human being, resulting in a semantically anomalous sentence.

(12) #Hirata-wa  Iida-o       nonda.
         Hirata-TOP Iida-ACC drank
         “#Hirata drank iida.”

#### Procedure

This experiment was conducted using E-Prime (Psychology Software Tools, Inc.) with an external mouse for participants' use in responding. Stimuli were presented to the participants in random order in the center of the computer screen. After a fixation mark (+) appeared in the center of the screen for 2000 ms, a transitive sentence was presented as a target sentence and participants were asked to judge whether it was semantically acceptable or unacceptable by pressing the left mouse button for “yes” or the right mouse button for “no.” Instructed to respond as quickly and accurately as possible, participants' reaction times were registered from the point where a transitive sentence was shown on the screen to the point when participants clicked the mouse to answer. Seven practice sentences were given before the start of the actual trial.

#### Data analysis

Analyses of variances (ANOVAs) were carried out for target sentences (48 correct sentences) both on reaction times and error rates, taking subject (*F1*) and item (*F2*) variables into consideration. There were two factors considered in this analysis: the scrambling factor (+/−scrambling) and the topicalization factor (+/−topicalization). With regard to the analysis of reaction times, only correctly judged target sentences were selected. The analyses of the reaction times were conducted in three steps: First, extremes among sentence reading in addition to correctness decision times (less than 500 ms and longer than 5000 ms) were regarded as missing values. Second, reaction times outside of 2.5 standard deviations at both the high and low ranges were replaced by boundaries indicated by 2.5 standard deviations from the individual means of participants in each category. Third, ANOVAs were performed.

### Results

#### Question accuracy

Table 1 shows the error rates for the correctness decision task.

**Table 1:** **Error rates (%) for target sentences**.

**Sentence Type**	***M***	***SD***
S_NOM_ O_ACC_ V (−scrambling, −topicalization)	0.67	2.21
S_TOP_ O_ACC_ V (−scrambling, +topicalization)	2.54	4.50
O_ACC_ S_NOM_ V (+scrambling, −topicalization)	2.21	4.38
O_TOP_ S_NOM_ V (+scrambling, +topicalization)	10.33	10.81

The main effect of both the scrambling factor [*F*_1(1, 47)_ = 25.23, *p* < 0.001; *F*_2(1, 11)_ = 19.12, *p* < 0.01] and the topicalization factor [*F*_1(1, 47)_ = 25.72, *p* < 0.001; *F*_2(1, 11)_ = 22.09, *p* < 0.001] were significant. In other words, the error rates of −scrambling conditions were lower than those of +scrambling conditions, and the error rates of −topicalization conditions were lower than those of +topicalization conditions. Moreover, there was a significant interaction between them [*F*_1(1, 47)_ = 17.84, *p* < 0.001; *F*_2(1, 11)_ = 9.98, *p* < 0.05]. Planned comparison with Ryan's Method revealed that the main effect of the topicalization factor was significant in the +scrambling condition [*F*_1(1, 94)_ = 43.44, *p* < 0.001; *F*_2(1, 22)_ = 31.16, *p* < 0.001] but not in the −scrambling condition [*F*_1(1, 94)_ = 2.31, *n.s*.; *F*_2(1, 22)_ = 1.51, *n.s*.]. Namely, the error rates of O_TOP_S_NOM_V were significantly higher than those of O_ACC_S_NOM_V, but there was no significant difference in error rates between S_TOP_O_ACC_V and S_NOM_O_ACC_V. In addition, the effect of the scrambling factor was significant in the +topicalization condition [*F*_1(1, 94)_ = 43.03, *p* < 0.001; *F*_2(1, 22)_ = 28.59, *p* < 0.001] but not in the −topicalization condition [*F*_1(1, 94)_ = 1.69, *n.s*.; *F*_2(1, 22)_ = 1.00, *n.s*.]. That is, the error rates of S_TOP_O_ACC_V were significantly lower than those of O_TOP_S_NOM_V, but there was no significant difference between S_NOM_O_ACC_V and O_ACC_S_NOM_V.

#### Reaction times

Table 2 shows the reaction times for the correctness decision task.

**Table 2:** **Reaction times (ms) for target sentences**.

**Sentence Type**	***M***	***SD***
S_NOM_ O_ACC_ V (−scrambling, −topicalization)	1410	364
S_TOP_ O_ACC_ V (−scrambling, +topicalization)	1414	416
O_ACC_ S_NOM_ V (+scrambling, −topicalization)	1512	450
O_TOP_ S_NOM_ V (+scrambling, +topicalization)	1626	425

The results revealed that the main effect of both the scrambling factor [*F*_1(1, 47)_ = 38.11, *p* < 0.001; *F*_2(1, 11)_ = 25.34, *p* < 0.001] and the topicalization factor [*F*_1(1, 47)_ = 7.19, *p* < 0.05; *F*_2(1, 11)_ = 7.36, *p* < 0.05] were significant. −scrambling sentences were responded to faster than +scrambling sentences, and −topicalization sentences were processed significantly faster than +topicalization sentences. Furthermore, the interaction between the two factors was significant [*F*_1(1, 47)_ = 6.09, *p* < 0.05; *F*_2(1, 11)_ = 5.96, *p* < 0.05]. Planned comparison with Ryan's Method demonstrated that the effect of the topicalization factor was significant in the +scrambling condition [*F*_1(1, 94)_ = 13.10, *p* < 0.001; *F*_2(1, 22)_ = 12.98, *p* < 0.01] but not in the −scrambling condition [*F*_1(1, 94)_ = 0.01, *n.s*.; *F*_2(1, 22)_ = 0.04, *n.s*.] and that the effect of the scrambling factor was significant both in the −topicalization condition [*F*_1(1, 94)_ = 7.91, *p* < 0.01; *F*_2(1, 22)_ = 6.27, *p* < 0.05] and in the +topicalization condition [*F*_1(1, 94)_ = 38.32, *p* < 0.001; *F*_2(1, 22)_ = 30.00, *p* < 0.001]. To put it more concretely, the reaction times for O_ACC_S_NOM_V sentences were faster than those for O_TOP_S_NOM_V sentences, but there was no significant difference between S_NOM_O_ACC_V sentences and S_TOP_O_ACC_V sentences.

### Discussion

The first experiment was conducted to examine the influence of syntactic complexity and frequency on the processing cost of Japanese transitive sentences. The results showed that there was no difference in processing cost between S_NOM_O_ACC_V and S_TOP_O_ACC_V, and their processing costs were lower than the processing cost of O_ACC_S_NOM_V, which in turn was lower than that of O_TOP_S_NOM_V. The results are summarized in (13a).

(13) a. Observed processing cost:
           S_NOM_O_ACC_V   =   S_TOP_O_ACC_V   <   O_ACC_S_NOM_V   <   O_TOP_S_NOM_V
        b. Syntactic prediction about processing cost:
           S_NOM_O_ACC_V   <   S_TOP_O_ACC_V   <   O_ACC_S_NOM_V   <   O_TOP_S_NOM_V
        c. Frequency-based prediction about processing cost
            S_TOP_O_ACC_V   <   S_NOM_O_ACC_V   <   O_ACC_S_NOM_V   =   O_TOP_S_NOM_V

Note that neither the syntactic prediction shown in (13b) nor the frequency-based prediction shown in (13c) agree with (13a). Yet, it is feasible to give a full account of (13a) through a unified analysis of syntax and frequency. First, in contrast to both predictions, the processing cost of S_TOP_O_ACC_V did not differ from that of S_NOM_O_ACC_V. This fact can be explained by supposing that the syntactic complexity of S_TOP_O_ACC_V is offset by its high frequency. Although S_TOP_O_ACC_V is syntactically more complex than S_NOM_O_ACC_V, S_TOP_O_ACC_V occurs more frequently than S_NOM_O_ACC_V. Thus, the processing cost of S_TOP_O_ACC_V pertinent to syntactic complexity is considered to be alleviated by its high frequency, as compared with S_NOM_O_ACC_V. Second, in opposition to the frequency-based prediction, the processing cost of O_TOP_S_NOM_V was higher than that of O_ACC_S_NOM_V. Recall that the difference in frequency between O_ACC_S_NOM_V and O_TOP_S_NOM_V is not large. Hence, the frequency difference may not be enough to incur a difference in processing cost. Consequently, only the syntactic effect has been observed, and the processing cost of O_TOP_S_NOM_V was thus higher than that of O_ACC_S_NOM_V.

In sum, the results of the first experiment are a mixture of the syntactic prediction and the frequency-based prediction. Thus, we can conclude that both syntactic complexity and frequency play a significant role in the processing of Japanese transitive sentences: S_NOM_O_ACC_V, S_TOP_O_ACC_V, O_ACC_S_NOM_V, and O_TOP_S_NOM_V. Next, we investigate the effects of discourse context on processing cost.

## Experiment 2

### Predictions

The purpose of the second experiment was to examine the interaction between syntactic complexity, frequency, and discourse context. Since we observed the interaction between syntax and frequency in the first experiment, we focused on the influence of information structure here. In the second experiment, we made allowances for the given-new distinction. It has been proposed by previous studies that scrambled objects are compatible with given information. Thus, given-new ordering is predicted to facilitate the processing of O_ACC_S_NOM_V.

### Method

#### Participants

A total of 64 Japanese students (28 male and 36 female) at Tohoku University took part in the second experiment. The average age was 21.5 years.

#### Materials

In order to measure the influence of given-new ordering, two-sentence passages, as shown in (14), were employed for the sentence correctness decision task. Each passage consisted of a context sentence, of which all were existential, and a target sentence, of which all were transitive. In order to make either the subject or the object in the target sentences given information, the referent of the subjects in the context sentences [e.g., *Morita* in (14a)] were reused in the directly succeeding target sentences^4^. In contrast, the proper nouns that did not appear in context sentences [e.g., *Shibata* in (14b)] belong to new information in the target sentences.

14. a. Kissaten-ni Morita-ga       iru.
         cafe-LOC  Morita-NOM is
         “There is Morita at the cafe.”
    b. Morita-ga       Shibata-o       matta.
         Morita-NOM Shibata-ACC waited.for
         “Morita waited for Shibata.”

The second experiment had a 2 × 2 × 2 factorial design, with the scrambling factor (+/−scambling), the topicalization factor (+/−topicalization), and the informational factor (given-new/new-given) as variables. Hence, there were eight experimental conditions, as shown in (15).

(15) Experimental Conditions:
a. S_NOM_/given O_ACC_/new V(−scambling, −topicalization, given-new)
b. S_NOM_/new O_ACC_/given V(−scambling, −topicalization, new-given)
c. S_TOP_/given O_ACC_/new V(−scambling, +topicalization, given-new)
d. S_TOP_/new O_ACC_/given V(−scambling, +topicalization, new-given)
e. O_ACC_/given S_NOM_/new V(+scambling, −topicalization, given-new)
f. O_ACC_/new S_NOM_/given V(+scambling, −topicalization, new-given)
g. O_TOP_/given S_NOM_/new V(+scambling, +topicalization, given-new)
h. O_TOP_/new S_NOM_/given V(+scambling, +topicalization, new-given)

Ninety-six sets of four correct two-sentence passages and 96 sets of four incorrect two-sentence passages were made and shuffled in Latin Square Design and split into eight lists of two-sentence passages. Twenty-four filler two-sentence passages were added to each list. As a result, each list comprises 48 incorrect, 48 correct, and 24 filler two-sentence passages. Each participant finished two lists, with a break between the first and the second list. The analyses were only conducted for the reaction times and error rates of the correct sentences. The length and frequency of each lexical material was controlled. Furthermore, none of the nouns or verbs were used repeatedly in order to prevent participants from encountering the same words in different two-sentence passages.

#### Procedure

As in the first experiment, the second experiment was conducted using E-Prime (Psychology Software Tools, Inc.) with an external mouse for participants' use in responding. The stimuli were presented to the participants in random order in the center of the computer screen. A context sentence and the target sentence associated with it were presented separately, each in its entirety. The participants read the context sentence at their own pace and pressed a button to advance to the presentation of the target sentence. They were asked to judge whether the target sentence was semantically acceptable or unacceptable by pressing the left mouse button for “yes” and the right mouse button for “no.” After each response to a target sentence, a fixation mark (+) appeared in the center of the screen for 2000 ms before moving on to the next stimulus. The reaction times for the target sentences were registered from the point of stimulus presentation on the screen to the point of participant response. Seven practice two-sentence passages were given to each participant prior to the commencement of the actual trial.

#### Data analysis

Analyses of variances (ANOVAs) were conducted on reaction times and error rates for target sentences (48 correct sentences), using the subject (*F1*) and item (*F2*) variables. There were three factors for our analysis: the scrambling factor (+/−scrambling), the topicalization factor (+/−topicalization), and the informational factor (given-new/new-given). Only correctly judged target sentences were used in the analyses of reaction times. First, extremes among sentence reading in addition to correctness decision times (less than 500 ms and longer than 5000 ms) were recorded as missing values. Second, reaction times outside of both the high and the low ranges of 2.5 standard deviations were replaced by those indicated from the individual means of participants by 2.5 standard deviations in each category.

### Results

#### Question accuracy

Table 3 summarizes the error rates for target sentences.

**Table 3:** **Error rates (%) for target sentences**.

**Sentence type**	***M***	***SD***
S_NOM_/given O_ACC_/new V (−scambling, −topicalization, given-new)	5.86	10.25
S_NOM_/new O_ACC_/given V (−scambling, −topicalization, new-given)	5.99	12.01
S_TOP_/given O_ACC_/new V (−scambling, +topicalization, given-new)	5.60	11.70
S_TOP_/new O_ACC_/given V (−scambling, +topicalization, new-given)	6.64	13.04
O_ACC_/given S_NOM_/new V (+scambling, −topicalization, given-new)	8.85	13.68
O_ACC_/new S_NOM_/given V (+scambling, −topicalization, new-given)	13.67	18.63
O_TOP_/given S_NOM_/new V (+scambling, +topicalization, given-new)	23.57	28.19
O_TOP_/new S_NOM_/given V (+scambling, +topicalization, new-given)	25.39	27.11

The significant main effects were observed for both the scrambling factor [*F*_1(1, 63)_ = 54.79, *p* < 0.001; *F*_2(1, 11)_ = 100.22, *p* < 0.001] and the topicalization factor [*F*_1(1, 63)_ = 33.27 *p* < 0. 001; *F*_2(1, 11)_ = 54.40, *p* < 0.001]. To put it more concretely, participants made more mistakes judging OSV sentences than they did SOV sentences. The error rates in the +topicalization conditions were higher than those in the −topicalization conditions. Moreover, the main effect of the informational factor was marginally significant [*F*_1(1, 63)_ = 7.62, *p* < 0.01; *F*_2(1, 11)_ = 3.64, *p* = 0.08], with given-new sentences processed more accurately than new-given sentences. Furthermore, there was a statistically significant interaction between the scrambling factor and the topicalization factor [*F*_1(1, 63)_ = 38.42, *p* < 0.001; *F*_2(1, 22)_ = 50.48, *p* < 0.01]. Planned comparison with Ryan's Method demonstrated that the effect of the topicalization factor was significant in OSV [*F*_1(1, 126)_ = 71.13, *p* < 0.001; *F*_2(1, 22)_ = 104.81, *p* < 0.001] but not in SOV [*F*_1(1, 126)_ = 0.01, *n.s*.; *F*_2(1, 22)_ = 0.02, *n.s*.]. In other words, there was a significant difference between O_ACC_S_NOM_V and O_TOP_ S_NOM_ V, but not between S_NOM_ O_ACC_ V and S_TOP_ O_ACC_ V. The effect of the scrambling factor was significant both in the −topicalization condition [*F*_1(1, 126)_ = 7.76, *p* < 0.01; *F*_2(1, 22)_ = 12.57, *p* < 0.005] and the +topicalization condition [*F*_1(1, 126)_ = 91.96, *p* < 0.001; *F*_2(1, 22)_ = 150.40, *p* < 0.001]. In other words, the error rates of OSV were higher than those of SOV.

#### Reaction times

The correct decision reaction times are shown below in Table 4.

**Table 4:** **Reaction times (ms) for target sentences**.

**Sentence type**	***M***	***SD***
S_NOM_/given O_ACC_/new V (−scambling, −topicalization, given-new)	1688	515
S_NOM_/new O_ACC_/given V (−scambling, −topicalization, new-given)	1822	565
S_TOP_/given O_ACC_/new V (−scambling, +topicalization, given-new)	1705	515
S_TOP_/new O_ACC_/given V (–scambling, +topicalization, new-given)	1748	558
O_ACC_/given S_NOM_/new V (+scambling, −topicalization, given-new)	1899	633
O_ACC_/new S_NOM_/given V (+scambling, −topicalization, new-given)	2141	865
O_TOP_/given S_NOM_/new V (+scambling, +topicalization, given-new)	2155	917
O_TOP_/new S_NOM_/given V (+scambling, +topicalization, new-given)	2193	807

There were significant main effects of the scrambling factor [*F*_1(1, 63)_ = 80.59, *p* < 0.001; *F*_2(1, 11)_ = 153.04, *p* < 0.001] and the informational factor [*F*_1(1, 63)_ = 22.11, *p* < 0.001; *F*_2(1, 11)_ = 2.52, *n.s*.]. The main effect of the topicalization factor [*F*_1(1, 63)_ = 4.69, *p* < 0.05; *F*_2_ = 1.96, *n.s*.] was significant only in the participant analysis. The interaction between the topicalization factor and the informational factor was significant [*F*_1__(1, 63)_ = 9.72, *p* < 0.01; *F*_2(1, 11)_ = 14.34, *p* < 0.01]. This interaction was statistically significant in OSV [*F*_1(1, 63)_ = 4.39, *p* < 0.05; *F*_2(1, 11)_ = 10.90, *p* < 0.01] and was marginally significant in SOV [*F*_1(1, 63)_ = 3.94, *p* = 0.051; *F*_2(1, 11)_ = 3.28, *p* = 0.09]. In addition, the effect of the informational factor was significant in O_ACC_S_NOM_V [*F*_1(1, 126)_ = 16.34, *p* < 0.001; *F*_2(1, 22)_ = 6.68, *p* < 0.05] although it was not in O_TOP_S_NOM_V [*F*_1(1, 126)_ = 0.40, *n.s*.; *F*_2(1, 22)_ = 0.45, *n.s*.]. In other words, O_ACC_S_NOM_V sentences were more strongly influenced by given-new ordering than O_TOP_S_NOM_V. Moreover, the scrambling factor and the topicalization factor were found to interact [*F*_1(1, 63)_ = 11.71, *p* < 0.005; *F*_2(1, 11)_ = 23.81, *p* < 0.001]. Planned comparison with Ryan's Method revealed that the effect of the topicalization factor was significant in OSV [*F*_1(1, 126)_ = 12.29, *p* < 0.001; *F*_2(1, 22)_ = 11.58, *p* < 0.005] but not in SOV [*F*_1(1, 126)_ = 0.47, *n.s*.; *F*_2(1, 22)_ = 0.78, *n.s*.]. In other words, there was a significant difference between O_ACC_S_NOM_V and O_TOP_S_NOM_V, but not between S_NOM_O_ACC_V and S_TOP_O_ACC_V. The effect of the scrambling factor was significant both in the −topicalization condition [*F*_1(1, 126)_ = 30.63, *p* < 0.001; *F*_2(1, 22)_ = 58.50, *p* < 0.001] and in the +topicalization condition [*F*_1(1, 126)_ = 87.57, *p* < 0.001; *F*_2(1, 22)_ = 169.42, *p* < 0.001]. In other words, SOV sentences were processed faster than OSV sentences.

### Discussion

The purpose of the second experiment was to explore the influence of given-new ordering on the processing cost of S_NOM_O_ACC_V, S_TOP_O_ACC_V, O_ACC_S_NOM_V, and O_TOP_S_NOM_V. First, it was demonstrated that there were interactions between the scrambling factor and the topicalization factor in both error rates and reaction times. There was no significant difference in processing cost between S_NOM_O_ACC_V and S_TOP_O_ACC_V, but their processing costs were lower than that of O_ACC_S_NOM_V, which was less difficult to process than O_TOP_S_NOM_V. This result is compatible with the first experiment and can be explained by the influence of syntactic complexity and frequency.

Second, the interaction between the informational factor and the topicalization factor was significant in reaction times. This is because given-new ordering alleviated the processing cost of O_ACC_S_NOM_V. This result is consistent with Kaiser and Trueswell's (2004) findings in that the processing costs of the scrambled sentences were reduced in a supportive context. Furthermore, this is compatible with Kuno's (1978) claim that the motivation for scrambling is to put the older information first and the newer information later in the sentence.

To sum up the above discussion in terms of information structure, given-new ordering had a greater impact on O_ACC_S_NOM_V than S_NOM_O_ACC_V, S_TOP_O_ACC_V, or O_TOP_S_NOM_V. What, therefore, is the cause of this gap? One possibility relies on the combination of the two functional constraints shown in (16) and (17).

(16) Markedness Principle for Discourse Rule Violations:
Sentences that involve marked (or intentional) violations of discourse principles are unacceptable. On the other hand, sentences that involve unmarked (or unintentional) violations of discourse principles go unpenalized and are acceptable.

(17) Information Flow Principle (IFP)
In principle, words in a sentence are arranged in such a way that those that represent old, predictable information come first, and those that represent new, unpredictable information come last.
         (Kuno, 1978, p. 54)

Let us consider the outcome of the second experiment in terms of (16) and (17). Firstly, S_NOM_O_ACC_V and S_TOP_O_ACC_V are not responsive to the IFP. There is no penalty for violating the IFP in SOV because SOV is an unmarked option/word order. Indeed, reaction times were not slowed down and the error rates did not become higher even in the inappropriate context (new-given condition). The given-new ordering is a desirable order for SOV, but it is not a mandatory requirement. Secondly, O_ACC_S_NOM_V is susceptible to IFP. Note that scrambling is a marked option. Therefore, O_ACC_S_NOM_V is penalized when it violates IFP. In fact, the processing costs of O_ACC_S_NOM_V become higher in the new-given condition. Thirdly, O_TOP_S_NOM_V is not sensitive to IFP. There were no significant differences in reaction times and error rates between the new-given and given-new conditions. Apparently, this seems to run counter to (16) because there appears to be no penalty for O_TOP_S_NOM_V when it violates the IFP despite being a marked word order. Yet, recall that O_TOP_S_NOM_V was processed very slowly even in the given-new condition. Indeed, given-new ordered O_TOP_S_NOM_V was processed as slowly as the new-given ordered O_ACC_S_NOM_V, leading to the conclusion that O_TOP_S_NOM_V was penalized even when it followed IFP. Indeed, the error rates of O_TOP_S_NOM_V were the highest of all the conditions. Thus, it is possible that O_TOP_S_NOM_V was not given supportive discourse contexts in the second experiment and that O_TOP_S_NOM_V was penalized even when it followed IFP because it requires different (e.g., more contrastive) discourse contexts for felicitous interpreting.

In sum, the Markedness Principle for Discourse Rule Violations is critical in explaining the results of the second experiment. We conducted a sentence comprehension experiment to see if there was an influence of given-new ordering on scrambling and topicalization. The results revealed that the processing cost of scrambling was mitigated in the given-new condition. However, the processing of topicalization was not facilitated by given-new ordering. This coincides with previous studies stating that the marked pattern occurs only in the licensing context, whereas the unmarked pattern is contextually unrestricted (Aissen, 1992; Kuno, 1995; Birner and Ward, 2009; Koizumi et al., 2014). Specifically, Birner and Ward (2009) pointed out that canonical word order can be permitted in a wide range of contexts whereas non-canonical word orders cannot be used without a licensing context. Considering Japanese scrambling in terms of this tendency, SOV can be used freely even when it violates the discourse principle of IFP because it is an unmarked option. In contrast, OSV is not permitted when it violates the IFP because it is a marked option.

## General discussion

In this study, we compared the effect of syntactic complexity, frequency, and discourse context on the processing of S_NOM_O_ACC_V, S_TOP_O_ACC_V, O_ACC_S_NOM_V, and O_TOP_S_NOM_V sentences in Japanese. In the first experiment, both syntactic complexity and frequency demonstrated an effect on the processing cost of each condition. To put it more concretely, the first experiment revealed that syntactically complex constructions are more difficult to process than syntactically simple constructions and that low-frequency constructions induce higher difficulty than highly frequent constructions do. Although the processing cost of O_ACC_S_NOM_V and O_TOP_S_NOM_V can be explained by the syntactic account, it is essential to take the frequency-based account into consideration so as to explain the processing cost of S_TOP_O_ACC_V. This is because the result concerning S_TOP_O_ACC_V sentences was a mixture of the syntactic prediction and the frequency-based prediction. Note that the processing costs of S_TOP_O_ACC_V that derive from syntactic complexity were neutralized by its high frequency. Thus, syntactic complexity affects the processing cost in tandem with frequency. In sum, the results of the first experiment demonstrated that both syntactic complexity and frequency affect the processing cost of S_NOM_O_ACC_V, S_TOP_O_ACC_V, O_ACC_S_NOM_V, and O_TOP_S_NOM_V.

In the second experiment, not only syntactic complexity and frequency but also discourse context had an influence on the processing cost of each condition. Specifically, the processing cost of O_ACC_S_NOM_V was facilitated by given-new ordering. Can this be taken as evidence that scrambling does not have a psycholinguistic reality? Our answer is “no.” Even in the given-new condition, the processing cost of the scrambled word order was higher than that of its canonical counterpart. In other words, information structure could not override the cost pertinent to scrambling. This indicates that some parts of the processing cost derive from syntactic complexity and they are robust enough to avert repair by the pragmatic factor. This supports the view that the syntactic structure-building process is primary and decoding of discourse structure is secondary in online processing (Kizach and Balling, 2013). However, it is conceivable that the processing cost of scrambling cannot be erased because of our artificial stimuli. In our experiment, the same nouns were presented twice in order to make the subject or the object given information. This design is unnatural because Japanese speakers prefer to use pro-drop to refer to the same referent again. Other factors like this may have had a negative influence on the processing of scrambling in our experiment. Further studies are needed in order to examine whether the syntax-based processing cost can disappear completely when an appropriate context is provided. Is it possible to erase the processing cost pertinent to the syntactic operation through discourse context? This question is beyond the scope of this paper and thus we leave it open to future research.

Next, it was revealed that Kuno's (1987) Markedness Principle for Discourse Rule Violations could provide an explanation of the results of the second experiment as a whole: SOV sentences are unmarked options, and as such were not penalized even when they violated IFP, O_ACC_S_NOM_V was penalized when it did not follow IFP due to its markedness, and O_TOP_S_NOM_V was always penalized in our experiment because appropriate context was not provided. Kuno (1973, p. 357) stated that “if a *non-subject* noun phrase is followed by *wa*, ordinarily only the contrastive interpretation results.” Moreover, McGloin (1990, p. 113) even maintained that O_TOP_S_NOM_V tends to have a contrastive meaning. Taken together, there seems to be a correlation between *WA*-marked objects and contrastiveness in O_TOP_S_NOM_V. Thus, O_TOP_S_NOM_V seems to be penalized without a contrastive context. One example of such a contrastive context is a multiple *wh*-question. Since a multiple *wh*-question sentence (18a) induces a pair-list answer, the speaker answers this question by using two O_TOP_S_NOM_V sentences in (18b). Note that (18b) includes a contrastive set, whose members are *isshoo* (Chapter 1) and *nishoo* (Chapter 2). Topicalization is a plausible grammatical option in (18b) because the first chapter is explicitly contrasted with the second chapter. Further discussion of the relationship between topicalization and contrastive context is beyond the scope of this paper.

(18) a. Dare-ga                nan-shoo-o       kaita-no?
         who-NOM           which-chapter   wrote-Q
         “Who wrote which chapter?”
        b. Isshoo-wa           Yamada-ga        kai-te,
         Chapter 1.TOP   Yamada-NOM  wrote-and
         nishoo-wa            Ishida-ga           kaita-yo.
         Chapter 2.TOP    Ishida-NOM     wrote-FP
         “Yamada wrote Chapter 1, and Ishida wrote Chapter 2
         (and I don't know about the other chapters).”

Another possible explanation for the processing cost of O_TOP_S_NOM_V points to the garden path effect. The parser cannot decide the grammatical function of the first NP in O_TOP_S_NOM_V until the second NP is processed because NP_TOP_ can be both the subject and the object. In such a locally ambiguous situation, there is an overall bias toward temporarily interpreting the first NP as the subject. However, the grammatical function of the first NP is not the subject but the object. Consequently, the parser needs to revise its interpretation of the first NP from the subject to the object when it encounters the second NP. This reanalysis may be the cause of the high processing cost of O_TOP_S_NOM_V. In addition, in the given-new condition, the referent of the NP_TOP_ in O_TOP_S_NOM_V is once mentioned in the preceding context. Under this context, our participants may be led to believe that NP_TOP_ is the subject when they encounter it because there is an interrelation between subjectivity and topicality according to Lambrecht (1996, p. 131). Thus, it is conceivable that the garden path effect caused by the subject-biased interpretation brought about the processing cost of O_TOP_S_NOM_V even in the given-new condition. In German, Meng et al. (1999) observed that even supportive contexts could not offset the garden path effect caused by an ambiguous scrambled order (OS-order). The parser tries to process ambiguous scrambled sentences by treating them as canonical order sentences at first.

In sum, our experiments revealed that syntactic complexity, frequency, and discourse context influenced the processing cost of S_NOM_O_ACC_V, S_TOP_O_ACC_V, O_ACC_S_NOM_V, and O_TOP_S_NOM_V. In other words, all three factors played a main role in processing each construction. The processing cost of syntactic complexity was offset by the frequency in the case of S_TOP_O_ACC_V. On the other hand, the syntactic cost of O_ACC_S_NOM_V was so strong that appropriate contexts could not erase it completely. Thus, it is possible that syntactic complexity and frequency first influence online parsing, while discourse context affects the human parser later. Furthermore, the garden path effect seems to have given rise to the high processing cost of O_TOP_S_NOM_V.

## Conclusion

We conducted two experiments in order to investigate the influence of syntactic complexity, frequency, and discourse context on the processing cost of S_NOM_O_ACC_V, S_TOP_O_ACC_V, O_ACC_S_NOM_V, and O_TOP_S_NOM_V. The first experiment demonstrated that both syntactic complexity and frequency had effects on the processing cost of each condition. In particular, the structure-building cost of S_TOP_O_ACC_V was offset by its high frequency. As a result, there was no difference in the processing cost of S_NOM_O_ACC_V and S_TOP_O_ACC_V, whose cost was lower than that of O_ACC_S_NOM_V, which showed a lower processing cost than O_TOP_S_NOM_V did. Therefore, it is reasonable to conclude that the human parser is affected by syntactic complexity and frequency simultaneously.

The second experiment was conducted to see whether there were interactions between syntactic complexity, frequency, and discourse context. Syntactic complexity was reflected in the processing cost. However, the cost of S_TOP_O_ACC_V was offset by its high frequency again. Furthermore, the effects of discourse context varied between the conditions. Specifically, the processing cost of O_ACC_S_NOM_V was lowered by given-new ordering, but the structure–building costs did not disappear. Moreover, the processing cost of the topicalization was not alleviated by given-new ordering. These distributions can be explained by the combination of the garden path effect and one of the functional constraints, the Markedness Principle for Discourse Rule Violations, as shown in (16).

## Author contributions

SI and MK contributed to the experimental design and the interpretation of data for the work. YS created the materials and conducted the experiments and statistical analyses. Based on the results, SI and MK wrote this article. MK financially supported this study.

## Funding

This work was supported by JSPS KAKENHI Grant Numbers 15H02603, 26580069.

### Conflict of interest statement

The authors declare that the research was conducted in the absence of any commercial or financial relationships that could be construed as a potential conflict of interest. The reviewer [KE] and handling Editor declared their shared affiliation, and the handling Editor states that the process nevertheless met the standards of a fair and objective review.

## Footnotes

^1^SR is not always easier to process than OR in every language. For example, Carreiras et al. (2010) reports that SR is harder to process than OR in Basque. This plausibly indicates that the processing cost is not solely determined by structural complexity, a point taken up in this paper subsequently.

^2^Under the hypothesis (7b), OASV may be syntactically ambiguous between (i) O [_VP_ ASV] and (ii) OA_i_ [_VP_ t_i_ SV] (in both of which the traces of the object are not represented here). Given the general assumption widely held in the sentence-processing literature that the human parser preferentially constructs simpler syntactic structures when input and grammar allow for multiple possibilities (see, e.g., Frazier and Fodor, 1978; Pritchett and Whitman, 1995; Gibson, 1998; Hawkins, 2004), the human parser is likely to construct the structure (i) rather than (ii).

^3^The relative frequencies of the four constructions in question cannot be accounted for solely by syntactic complexity. Suppose, as is plausible, that simpler structures are generally preferred to more complex ones. Then the higher frequencies of SOV sentences (StopOV, SnomOV) than OSV (OtopSV and OaccSV) sentences may be at least partially attributable to the simpler syntactic structures of the former than the latter, However, syntactic complexity alone cannot explain the higher frequency of StopOV than SnomOV. Recall that we follow a standard assumption that StopOV is syntactically more complex than SnomOV, which would lead to the expectation that the former should be less frequently used than the latter, contrary to the fact. A reviewer pointed out a possibility that Stop may occupy the same syntactic position as Snom, and hence StopOV and SnomOV may be comparable in terms of syntactic complexity. If that turns out to be the case, we would then expect that StopOV is as frequently produced as SnomOV as far as syntactic complexity is concerned, again an incorrect prediction. Thus, the higher frequency of StopOV than SnomOV calls for an explanation other than (or in addition to) syntactic complexity. Although it is beyond the scope of the present paper to propose a fully explanatory account, the relatively higher frequency of StopOV may be attributable to the following two factors. First, subject tends to be topicalized because of the prominence of its referent as a result of properties such as agency, animacy, concreteness, prototypicality, and/or prior mention in the discourse. Second, sentences with a non-topicalized nominative subject are used to describe an event in neural context, whereas sentences with a topicalized subject are used when the referent of the subject is discourse-given. In natural discourse, the latter tend to be used more frequently than the former, because they contribute to increase discourse cohesion by establishing and/or maintaining a discourse topic. Hence StopOV is more frequently used than SnomOV.

^4^The repetition of the same noun is a bit unnatural, but if a zero pronoun is used, we cannot measure the processing cost of OSV because SOV is the only possible interpretation. Moreover, if an overt pronoun were chosen for the experiment, it would give rise to overly strong priming effects caused by using the same pronoun repeatedly. Furthermore, it is not as awkward to repeat the same noun twice in Japanese as it is in English.
